# Melanin as a Virulence Factor in Different Species of Genus *Paracoccidioides*

**DOI:** 10.3390/jof6040291

**Published:** 2020-11-17

**Authors:** Elúzia C. P. Emidio, Martha E. Urán J., Leandro B. R. Silva, Lucas S. Dias, Mariana Doprado, Joshua D. Nosanchuk, Carlos Pelleschi Taborda

**Affiliations:** 1 Instituto de Ciencias Biomedicas, Departamento de Microbiologia, Universidade de Sao Paulo, Sao Paulo 05508-000, Brazil; eluziaemidio@gmail.com (E.C.P.E.); leandrobr87@usp.br (L.B.R.S.); lucasdias@usp.br (L.S.D.); marianadoprado@yahoo.com.ar (M.D.); 2 Instituto de Medicina Tropical de Sao Paulo, Laboratorio de Micologia Medica/LIM53, Departamento de Dermatologia, Faculdade de Medicina, Universidade de Sao Paulo, Sao Paulo 05403-000, Brazil; martha.uran@udea.edu.co; 3 Department of Microbiology and Parasitology, School of Medicine, Universidad de Antioquia, Medellín 050010, Colombia; 4 Departments of Medicine (Division of Infectious Diseases) and Microbiology and Immunology, Albert Einstein College of Medicine, Bronx, NY 10461, USA; josh.nosanchuk@einsteinmed.org

**Keywords:** *Paracoccidioides*, *Paracoccidioides* spp. melanin, virulence factor, paracoccidioidomycosis

## Abstract

Paracoccidioidomycosis (PCM) is a granulomatous systemic mycosis caused by the thermo-dimorphic fungi of the genus *Paracoccidioides*. Melanin production by fungi can affect their pathogenesis and virulence. This study evaluates the production of melanin by different isolates of genus *Paracoccidioides* and examines how the presence of this polymer affects yeast cell phagocytosis, as well as laccase enzyme production. The results obtained showed that the isolates of genus *Paracoccidioides*: *P. lutzii* (Pb01, Pb66, ED01, Pb1578, and Pb8334), *P. restrepiensis* (PS3-Pb60855), *P. brasiliensis* (S1-Pb18), and *P. americana* (PS2-Pbcão) produce melanin in the presence of L-3,4-dihydroxyphenylalanine (L-DOPA). Phagocytosis assays were carried out with peritoneal macrophages from C57Bl/6 mice that were challenged with Pb18, Pb60855, and Pb01. We observed that melanin interferes with phagocytosis in the presence or absence of complement or heat-inactivated serum. This article confirms that different species of the genus *Paracoccidioides* produce melanin in different magnitudes and that the polymer functions as a virulence factor.

## 1. Introduction

Paracoccidioidomycosis (PCM) is a systemic mycosis caused by the thermally dimorphic fungi of the *genus Paracoccidioides*. The infection is initiated with the inhalation of propagules and conidia from the mycelial phase, which are deposited into pulmonary alveoli, where they undergo conversion to the yeast phase, which is the pathogenic form [1]. From this point, the fungus can spread hematogenous and/or via the lymphatics to any part of the affected host [2,3,4,5]. PCM affects both immunocompromised and immunocompetent individuals [6,7] and is most prevalent in rural populations of subtropical areas from the south of Mexico to the north of Argentina. Brazil is the country with the highest number of registered cases. However, the prevalence and incidence of this disease are difficult to estimate, particularly as PCM is a neglected disease that lacks a prevention program, and it is not a reportable disease [4,6]. However, reviews estimate the annual incidence from 1 to 3.7 cases per 100,000 people in Brazil (reviewed by Queiroz-Telles et al., 2017) [8].

Although variability among isolates has been recognized for decades, advanced molecular techniques have revealed that “*P. brasiliensis*” isolates from different regions have significant genetic variability and that they are, in fact, comprised of a complex of cryptic species (S1, PS2, PS3, and PS4) [1,7,9,10]. In 2014, Teixeira et al. described a formal new species within the genus *Paracoccidioides*, which has been named *Paracoccidioides lutzii*. This species is endemic mainly in the North and Central-West regions of Brazil (Rondônia, Mato Grosso, Goiás, etc.) [7,11,12]. Comparative genomic and proteomic studies between *P. brasiliensis* complex and *P. lutzii* have revealed significant differences that may influence the diagnosis and treatment of PCM [7,13,14]. Based on detailed molecular analyses, there are currently five recognized *Paracoccidioides* species, referred to as *Paracoccidioides brasiliensis sensu stricto* for S1, *P. americana* for PS2, *P. restrepiensis* for PS3, *P. venezuelensis* for PS4, and *P. lutzii* isolates [15].

Melanins are ubiquitous pigments in nature, and they are widely distributed in all biological kingdoms [16]. Melanins are amorphous chemical structures formed by the oxidative polymerization of phenolic and/or indole compounds [17]. In fungi, two main classes of melanin can be found, depending on their biosynthetic pathway: The polysaccharide-synthetase pathway, with the formation of DHN-melanins, dihydroxynaphthalene melanins, synthesized from substrates that are produced endogenously; and the phenoloxidases or laccases pathways, melanins derived from phenolic compounds like L-3,4-dihydroxyphenylalanine (L-DOPA), an exogenous precursor that undergoes oxidation catalyzed by laccase [16,17].

*Paracoccidioides* isolates have been shown to produce melanin, and the pigment is associated with the pathogenesis of PCM. [16,18,19,20]. Previous descriptions showed that *P. brasiliensis* (Pb18) melanized cells are more resistant to attack by alveolar and peritoneal macrophages and are less susceptible to antifungal drugs, especially amphotericin B. In a murine intratracheal infection model, the challenge with melanized yeast cells resulted in higher lung colony forming units compared to infection with non-melanized yeast. In addition, melanized cells were more resistant to attack by the oxidative stress of macrophages, thus confirming that melanized cells show greater resistance to phagocytosis [18,19].

The present study aimed to evaluate the production of melanin by different isolates of the genus *Paracoccidioides*, with a focus on *P. lutzii* isolates, and to assess the effects of melanization on potentially pathogenic processes in these isolates. We compared different isolates to determine if variations in melanization could be a predictor for virulence and affect host-pathogen interactions.

## 2. Materials and Methods

### 2.1. Fungal Strains

*Paracoccidioides brasiliensis complex* strains are represented by: *P. restrepiensis* for PS3 (isolate ATCC Pb60855), *P. brasiliensis sensu stricto* for S1(isolates Pb18), *P. americana* for PS2 (isolate Pbcão) and *P. lutzii* (isolates Pb01, Pb66, ED01, Pb1578, and Pb8334) [15,21]. *P. venezuelensis* strains were not available, and were not included in our analyses. *Candida albicans* (Ca12A) and *Cryptococcus neoformans* (ATCC 28957) were used as controls and cultivated as previously shown by da Silva et al. (2006). All strains were maintained in the culture collection of the Laboratory of Pathogenic Dimorphic Fungi, Department of Microbiology, Institute of Biomedical Sciences, University of São Paulo.

### 2.2. Growth Conditions

The yeasts of *Paracoccidioides* spp. were grown on BHI Agar (BD Difco™), supplemented with asparagine (Sigma-Aldrich, St. Louis, MO, USA) and thiamine (Sigma-Aldrich) for seven days at 37 °C and transferred to the Mcveigh-Morton chemically defined solid medium (SMVMM) modified by Restrepo and Jiménez (1980), incubated for seven days at 37 °C. Then, they were transferred to the chemically defined liquid medium-SMVMM, also prepared according to Restrepo and Jiménez (1980) (pH 5.5), in the presence or absence of 1,0 mM L-DOPA (L-3,4-Dihydroxyphenylalanine; Sigma-Aldrich) at 37 °C with shaking (150 RPM) and the yeasts were used after 10 or 15 days of growth [16,22]. The cultures were cultivated in the dark to prevent photopolymerization of L-DOPA. Isolates Pb18 and Pb60855 were used as controls, since we previously demonstrated that both produce melanin [23]. The mycelial phase of the isolates Pb60855, Pb18 and Pb01 were maintained in SMVMM solid medium, at 18 °C and 68% humidity. Cell viability was determined by staining with Trypan Blue 0.4% (Sigma-Aldrich) and was higher than 90% for all cultures utilized.

### 2.3. Growth Curves and Cell Melanization

Growth curves were performed for *P. lutzii* isolates (Pb01, Pb66, ED01, Pb1578, Pb8334) as described by Uran et al. (2008) [16]. Yeast cells (1.5 × 10^6^ yeasts/mL) of each isolate were inoculated in SMVMM liquid medium (pH 5.0) in the absence or presence of 0.1 mM and 1.0 mM L-DOPA. Fractions from each culture on days 3, 6, 9, 12, and 15 were collected and evaluated for growth and melanization by determining the number of melanized and non-melanized cells by visualization and counting by optical microscopy in a Neubauer camera. The frequency of melanized cells was reported as an average of three separated experiments.

### 2.4. Immunofluorescence Assay

**Figure 1:**
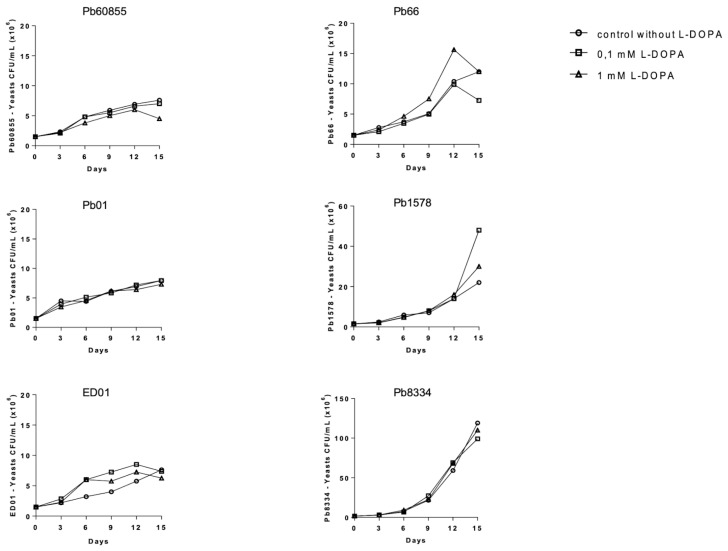
Growth of *Paraccocidioides* without or with L-DOPA (0.1 and 1 mM). The growth of the different isolates of *Paracoccidioides* was measured by CFU determinations at 3, 6, 9, 12, and 15 days of cultivation at 37 °C. *P. restrepiensis* (Pb60855), *P. lutzii* (Pb66, Pb01, Pb1578, ED01, and Pb8334). The results presented refer to one experiment from three independent experiments.

The technique was performed as described by Youngchim et al., 2004 [24]. Briefly, the yeasts cultured with 1.0 mM L-DOPA, for 10 days at 37 °C, with shaking (150 RPM), were heat fixed on a glass slide by cytocentrifugation and were blocked overnight with 5% BSA (Bovine Serum Albumin) blocking solution in PBS at 4 °C. Monoclonal antibodies (Mab) against melanin of *Paracoccidioides* Pb60855 (Mab 8C3, IgG1) and against melanin of *C. neoformans* (Mab 6D2, IgM) [20] were used. These were diluted in PBS, applied, and incubated for 1 h at 37 °C. After washing with PBS, the isotype-specific secondary antibodies were applied and incubated for 1 h at 37 °C. Anti-immunoglobulin antibodies Alexa Fluor^®^ 488 *goat* anti-mouse IgM and IgG (Life Technologies, Grand Island, NY, USA) were used as secondary antibodies, anti-IgG, when Mab8C3 was used, and anti-IgM, when Mab6D2 was used. The samples were analyzed by EVOS FL-AMG and NIKON ECLIPSE TI immunofluorescence microscopes with 400× and 600× magnification (Figure 1).

### 2.5. Laccase Activity

The colorimetric analysis of laccase activity was performed according to the protocol described by Da Silva et al. [18] with minor modifications. The yeast cells were initially cultured in liquid medium BHI (BD Difco^TM^) at 37 °C, with shaking (150 RPM). After seven days of growth, they were washed three times with PBS and suspended in liquid medium SMVMM with glucose. Yeasts were incubated for 48 h at 37 °C, with shaking (150 RPM). The cells were then washed three times with glucose-free liquid medium SMVMM, suspended in the same medium, and incubated for 48 h under the same conditions as described above. After the incubation, the cells were washed with PBS, and the cell number was adjusted to 10^8^ cells/mL for each isolate, and this suspension was centrifuged. The supernatant was discarded, and the pellets were suspended in 900 μL of 0.1 M sodium acetate buffer (pH 5.0) and 100 μL of a sodium acetate buffer solution containing 10 mM ABTS [2,2′-azino-bis (3-ethylbenzthiazoline-6-sulfonate)] (Sigma-Aldrich) for 2 h. The cells were centrifuged, and the supernatant was read at 420 nm in a spectrophotometer. *C. albicans* 12A and *C. neoformans* ATCC28957 strains were used as negative and positive controls for laccase activity, respectively.

### 2.6. Phagocytosis Assay

Peritoneal macrophages from 8 week old C57Bl/6 male mice were obtained by peritoneal lavage performed with 10mL of 0.9% NaCl. The use of mice for this work was approved by the Ethics Committee on Animal Experiments of USP. The ethical approval information is registered on the number 94, page 22 of book 3, approved by University of Sao Paulo (USP) Ethics Committee on Animal Experiments on 27 August 2014. The macrophages were counted on a hemocytometer, adjusted to a concentration of 1 × 10^6^ cells/mL in RPMI medium, with 10% fetal bovine serum (FBS) and 1% Pen-Strep. 0.5 mL of this suspension was transferred to 24-well cell culture plates. Cells were incubated at 37 °C with 5% CO_2_ for 2 h to allow adherence of the macrophages to the plaque. Then, the medium was removed, and a fresh medium with 40 ng/mL IFN-γ (BD-Pharmingen, San Diego, CA, USA) was added to promote macrophage activation, and the cells were incubated for an additional 16 h at 37 °C with 5% CO_2_. After this time, the macrophages were challenged with melanized or non-melanized yeasts of Pb01, Pb60855, and Pb18 isolates in a ratio of 10:1 (macrophage: yeast) (4 × 10^4^ yeasts per well). Yeasts were pre-labeled with 100 μg/mL FITC for 1 h at 4 °C, washed twice with 0,9% NaCl and suspended in RPMI medium with 10% FBS. After 6 h of co-culture, the macrophages were washed twice with PBS, and the cells were desalted and transferred to tubes, centrifuged for 10 min at 400× *g* at 4 °C, and the pellets were labeled with anti-F4/80 monoclonal antibody Anti-mouse F4/80 Antigen APC (eBioscience, San Diego, CA, USA) for 30 min at 4 °C. After labeling, the cells were washed twice in PBS, suspended in 200 μL of PBS plus 1% fetal bovine serum (FBS), and analyzed immediately on FACSCalibur (Becton Dickinson, Franklin Lakes, NJ, USA) cytometer. Data were analyzed using the FlowJo software program (Star Tree, Ashland, OR, USA). The procedure described above was performed in duplicate. Controls included untreated yeast (yeasts), yeast treated with complement factors derived from complete filtered serum from healthy mice (yeast + C’), and yeast treated with heat-inactivated serum (yeast + IS).

To distinguish between internalized and surface-bound yeasts (yeast of *P. lutzii* and *P. brasiliensis* labeled with FITC), trypan blue (TB) (Trypan Blue, 250μg/mL, Sigma Aldrich) was used to quench the green fluorescence of the surface of macrophages. The TB technique was performed as described by Busetto et al., 2004, with minor modifications [25]. Phagocytosis assays were performed as described above, and adherent/ingested cells were measured using FLAC and FL4 channels of the FACSCalibur cytometer. The cell suspensions were then treated in an ice bath with 0.1 mL of a prepared TB solution in 0.1 M citrate buffer, pH 4.0, thereby reducing the pH of the samples and optimizing the fluorescence quenching effect by TB. After 1 min incubation in an ice bath, the samples were again analyzed. Macrophages labeled with anti-F4/80 monoclonal antibody were filtered, and the FL1 and FL3 channels were used to discriminate ingested yeasts (green fluorescence, FL1) from adherent yeasts (red fluorescence, FL3).

### 2.7. Statistical Analysis

All of the experiments performed in this paper were done in triplicate. Quantitative analyzes were performed using GraphPad Prism version 5.0 (GraphPad Inc., San Diego, CA, USA), and analysis of variance (ANOVA) was performed following Tukey’s post-test. Results were considered significant when *p* < 0.05.

## 3. Results

### 3.1. Influence of L-DOPA on the Growth and Melanization of Yeasts in Liquid Medium among Paracoccidioides spp.

**Figure 2:**
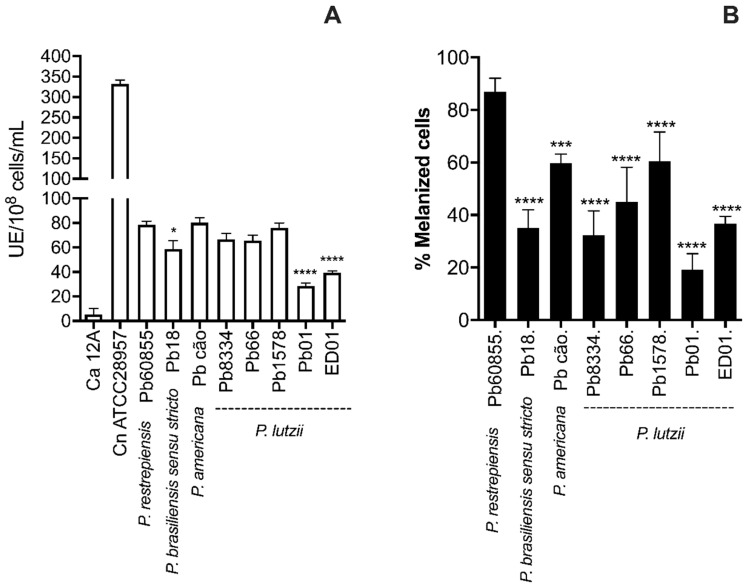
Pigmentation and laccase activity of *Paracoccidioides* isolates. Laccase activity was determined by colorimetric analysis (**A**). Percentages of melanized yeasts in medium with 1 mM L-DOPA, 37 °C, after 15 days of incubation (**B**) as determined by optical microscope visualization and counting. *C. albicans* 12A (Ca 12A) and *C. neoformans* (Cn ATCC28957) were used as negative and positive controls, respectively. The statistical representative differences were analyzed by one-way ANOVA followed by Tukey’s post-test, where * *p* <0.05, *** *p* < 0.001, and **** *p* < 0.0001 in comparison between the Pb60855 and the all others *Paracoccidioides*. The data represent the mean of results from three experiments.

**Figure 3:**
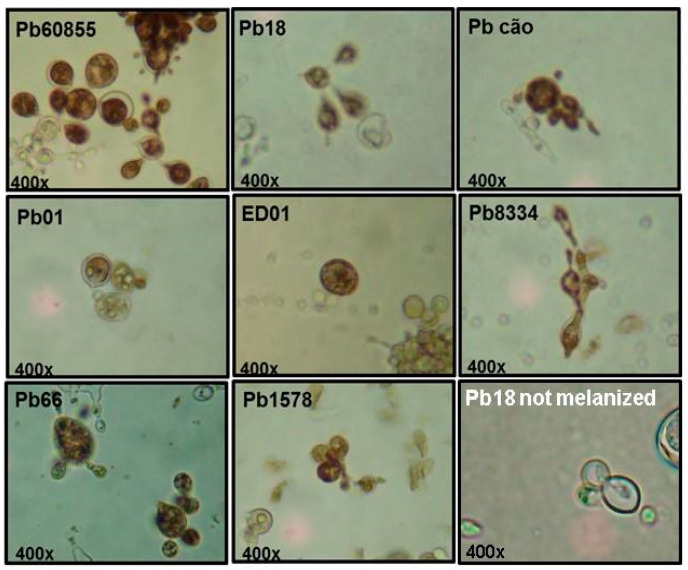
A panel of *Paracoccidioides* spp. yeast. Pb60855, Pb18, and Pbcão and *P. lutzii* (Pb01, ED01, Pb8334, Pb66, and Pb1578) were cultured in SMVMM liquid medium with L-DOPA, 37 °C, 150 RPM, for 15 days and then observed under light microscopy, 400× magnification. Pb18 “not melanized” represents Pb18 cultivated in the absence of L-DOPA.

To evaluate the influence of L-DOPA on the growth of *P. lutzii* yeast cells and the melanization profile, we assessed the impact of including L-DOPA (0.1 or 1 mM) in the medium using *P. restrepiensis* (*P. brasiliensis*—PS3—Pb60855) a control for comparison with *P. lutzii* isolates (Pb01, ED01, Pb66, Pb1578, and Pb8334). In Figure 1, we show that different concentrations in L-DOPA (0.1 and 1 mM) did not influence the growth of isolates of *Paracoccidioides* spp, as determined by CFU. Melanization was greatest for most isolates at day 15 (Figure 2). We subsequently tested additional isolates of *P. brasiliensis* (Pb18—S1) and *P. americana* (Pbcão—PS2), and these similarly melanized. For all isolates of the four species, the addition of the phenolic substrate led to the growth of blackened yeast, with pigmentation in the cytoplasm and cell wall (Figure 3).

### 3.2. Analysis of Percentage of Melanization and Enzymatic Activities

The percentage of melanized yeast cells at 15 days of incubation in a medium containing 1 mM L-DOPA ranged from 19% for Pb01 to 87% for Pb60855 (Figure 2B). Marked variation of cell melanization percentages also occurred within the same species as demonstrated with *P. lutzii* Pb01, with only 28% of cells melanizing compared to pigmentation in 65% of Pb1578. However, *P. brasiliensis sensu stricto* Pb18 also only melanized 34% of the time. Pb01 and ED01 had a quarter of melanized cells in comparison with other isolates (Figure 2B).

Laccase activities were also determined (Figure 2A), which also showed significant variations between species and isolates. *P. lutzii* Pb01 had the lowest laccase activity, and this correlated with the isolate having the lowest percentage of melanized yeast.

### 3.3. Immunofluorescence Assay (IF)

**Figure 4:**
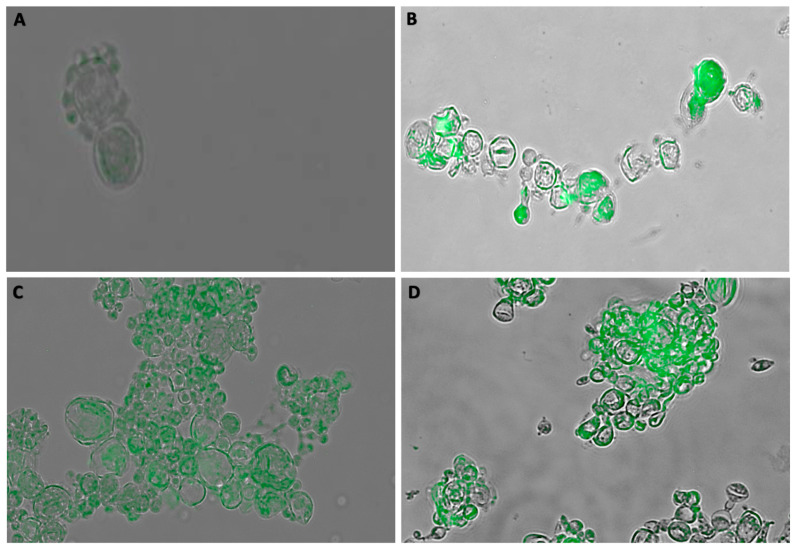
Immunofluorescences of *Paracoccidioides* yeast cultivated in the presence of 1 mM L-DOPA. (**A**) As a control, Pb60855 was incubated without Mab8C3, but with a fluorophore-labeled secondary antibody. The green fluorescence is indicative of the binding of Mab8C3 monoclonal antibody to melanin in Pb60855 (**B**), Pb18 (**C**), and Pb01 (**D**). The experiment was performed three times with similar results.

Immunofluorescence assays were performed with *Paracoccidioides* spp. yeast cells were grown in medium containing 1 mM L-DOPA. Two monoclonal antibodies (Mabs) that recognized melanin: IgG1 Mab8C3 (produced against *Paracoccidioides restrepiensis* melanin ghosts) [19] and IgM Mab6D2 (produced against *Cryptococcus neoformans* melanin ghosts) [26]. All isolates tested were recognized by both Mabs, and the fluorescence labeling profiles of the melanized cell wall were similar. Figure 4 depicts the binding of melanin by Mab8C3.

### 3.4. Phagocytosis Assay

**Figure 5:**
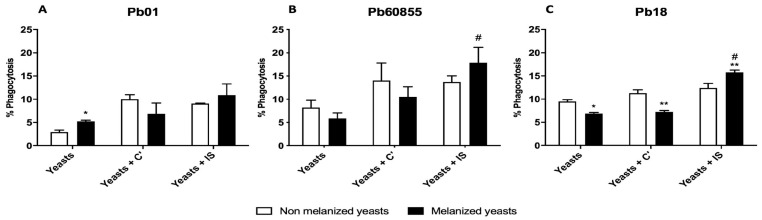
Pb01 (**A**), Pb18 (**B**), and Pb60855 (**C**) were co-cultured with macrophages in standard medium (Yeasts), medium with complement (Yeasts + C’), or medium with heat-inactivated serum (Yeasts + IS). Melanized yeast represents yeast cultured in the presence of L-DOPA for 15 days. The statistical representative differences were analyzed by one-way ANOVA followed by Tukey’s post-test, where * *p* < 0.05 and ** *p* < 0.01 in comparison between the non-melanized and melanized yeasts and # *p* <0.05 in comparison melanized yeasts of (Yeasts + C’) and (Yeasts + IS). The data represent the mean of results from three experiments.

To analyze the role of melanin in the virulence of different *Paracoccidioides* spp. isolates, phagocytosis assays were performed using Pb18, Pb60855, and Pb01 yeast isolated from media with or without L-DOPA, pretreated, or not with complete or heat-inactivated serum (Figure 5). Cultivation conditions did not significantly change the rates of phagocytosis of Pb01 in the presence of complement or heat-inactivated serum; however, we observed a significant increase in phagocytosis of yeast isolated from a medium with L-DOPA compared to control medium. In contrast, the culture of Pb18 in L-DOPA significantly reduced phagocytosis with or without complement, but L-DOPA treated cells were more avidly phagocytosed by macrophage in the presence of heat-inactivated serum. There was a trend toward reduced phagocytosis of L-DOPA exposed Pb60855 with or without complement, as well as a trend toward higher uptake with inactivated serum compared to yeast from the standard medium. Heat-inactivated serum significantly increased phagocytosis compared to complement treated Pb60855 L-DOPA exposed yeast.

## 4. Discussion

Paracoccidioidomycosis is classified as one of the main endemic systemic mycoses in Latin American countries, and it shares several clinical features with other mycoses, such as Histoplasmosis, North American Blastomycosis, and Coccidioidomycosis, as well as with other infections caused by intracellular pathogens, such as *Mycobacteria* and *Leishmania* spp. [6,27]. With the identification of the complex of cryptic species of the genus *Paracoccidioides* and with the description of *P. lutzii*, many questions have arisen regarding possible differences in the clinical forms of PCM. Some comparative studies of genomics and proteomics, between *Paracoccidioides* spp. and *P. lutzii*, have revealed significant differences that may influence the clinical manifestations, diagnosis, and treatment of PCM [7]. In this context, comparative studies aiming to identify differences among *Paracoccidioides* species, such as the characterization of virulence factors, can broaden the understanding of the different clinical manifestations observed and deepen our knowledge about PCM.

The production of melanin by several fungi is an important virulence factor, and the polymer can interfere with various mechanisms affecting pathogenesis. The process of melanization of *Paracoccidioides* species was first documented in *P. restrepiensis* (Pb60855) conidia and yeasts by Gomez et al. (2001) [28]. In 2006, Da Silva et al. showed that melanized cells from *P. brasiliensis* (Pb18) are more resistant to attack by alveolar and peritoneal macrophages, and are less susceptible to antifungal drugs, especially Amphotericin B [18]. Using a murine model, Da Silva et al. (2009) demonstrated increased fungal burdens in infections with melanized Pb18 yeast compared with non-melanized yeasts [13]. Urán et al. (2011) showed that melanin from *P. restripiensis* (Pb60855) is an antigenic molecule capable of inducing antibody production, which resulted in the generation of IgG and IgM isotype monoclonal anti-melanin antibodies [20]. Since both isolates have been used to prove melanin production by the *Paracoccidioides* genus and demonstrate that the pigment influences the pathogenicity of the fungus, we utilized them primarily as internal controls for the study of the other isolates. Our work focused on *P. lutzii* Pb01 as this isolate has been extensively characterized and is the exemplar of the species.

Our study sought to identify differences in the melanization process among different isolates from *Paracoccidioides*, covering the most relevant clinical species. We observed all isolates melanized, but the rate and degree of melanization differed. This is highlighted by the findings with the *P. lutzii* isolates examined (Pb01, ED01, Pb66, Pb1578, and Pb8334). The pigmentation of the yeast cells was confirmed as melanin by immunofluorescence microscopy using the melanin specific Mabs derived from *P. brasiliensis* melanin (Mab 8C3-IgG) and *C. neoformans* melanin (Mab 6D2-IgM) [20,29]. Regarding laccase, we observed that the isolates of the genus *Paracoccidioides* tested displayed widely variable enzyme activities. Notably, certain isolates generated similar laccase profiles compared to the *C. neoformans* control, which is well described as a high producer of laccase [30,31]. The differences in laccase activity were most striking amongst the *P. lutzii* isolates, ranging from 28 UE/10^8^ cells/mL in Pb01 to 78 UE/10^8^ cells/mL in Pb1578. Therefore, laccase levels are not an indicator of species, but it could be a correlate marker in relation to the virulence factors *per se* of each strain. Although laccase levels correlate well with the melanization percentages of most strains, they were discordant for *P. brasiliensis sensu stricto* Pb18 and *P. lutzii* Pb8334, which had relatively high activities of laccase, but lower levels of cell pigmentation. Nevertheless, the correlation between laccase activity and melanin production is well known, with laccase being the enzyme that catalyzes the formation of this metabolite [32]. However, subinhibitory concentrations of miltefosin, an analog of alkylphospholips that has antifugal activity, increased melanin production by yeast of *Paracoccidioides* ssp [33]. Possibly, molecular studies could clarify and establish a more consistent correlation between enzymatic activity and the melanization profile presented for each isolate.

To evaluate the role of melanin in the virulence of *Paracoccidioides* isolates, we performed phagocytosis assays. Initially, we observed that *P. brasiliensis sensu stricto* Pb18 cultivated with L-DOPA are less phagocytized compared to non-melanized yeasts, which corroborated the data in the literature [18]. However, the *P. lutzii* (isolate Pb01) grown with L-DOPA showed an increased rate of phagocytosis, which was not expected. *P. restrepiensis* isolate Pb60855 showed a different profile with no significant difference between melanized and non-melanized yeast cells. Isolates of *P. brasiliensis* (Pb18) and *P. restrepiensis* (Pb Pb60855), which have been previously studied, were used for comparisons with *P. lutzii*. Cultivation in the presence of L-DOPA seemed to protect the fungus against phagocytosis in the presence of complement factors, but this was only significant for Pb18. In contrast, heat-inactivation of serum increased phagocytosis of cells from cultures with L-DOPA. These data suggest that the interactions of melanin on the fungal cell surface with immune cells and components of serum are complex. Future in vivo studies may more clearly demonstrate the import of melanin in PCM.

In general, our results confirm that all of the examined *Paracoccidioides* species can produce melanin, albeit to different degrees. *Paracoccidioides* melanization requires the presence of a phenolic substrate, such as L-DOPA, and pigmentation is associated with the production and levels of activity of laccase. We also reveal marked differences in interactions of *Paracoccidioides* with and without cultivation in L-DOPA with primary macrophages, and the interactions varied among the species tested. Finally, the disparate findings amongst the *P. lutzii* isolates examined indicate that the *P. lutzii* isolate Pb01, which is used frequently to study pathogenicity, should not be considered as a reference isolate for the species.
